# Assessing the Effect of Humic Substances and Fe(III) as Potential Electron Acceptors for Anaerobic Methane Oxidation in a Marine Anoxic System

**DOI:** 10.3390/microorganisms8091288

**Published:** 2020-08-24

**Authors:** Sigrid van Grinsven, Jaap S. Sinninghe Damsté, Laura Villanueva

**Affiliations:** 1 Department of Marine Microbiology and Biogeochemistry, NIOZ Royal Netherlands Institute for Sea Research, Utrecht University, 1797 SZ ’t Horntje, Texel, The Netherlands; jaap.damste@nioz.nl (J.S.S.D.); laura.villanueva@nioz.nl (L.V.); 2 Department of Earth Sciences, Faculty of Geosciences, Utrecht University, 3584 CB Utrecht, The Netherlands

**Keywords:** ANME-1, anaerobic methane oxidation, Black Sea, AQDS, Fe(III), humic substances

## Abstract

Marine anaerobic methane oxidation (AOM) is generally assumed to be coupled to sulfate reduction, via a consortium of anaerobic methane-oxidizing archaea (ANME) and sulfate-reducing bacteria (SRB). ANME-1 are, however, often found as single cells, or only loosely aggregated with SRB, suggesting they perform a form of AOM independent of sulfate reduction. Oxidized metals and humic substances have been suggested as potential electron acceptors for ANME, but up to now, AOM linked to reduction of these compounds has only been shown for the ANME-2 and ANME-3 clades. Here, the effect of the electron acceptors anthraquinone-disulfonate (AQDS), a humic acids analog, and Fe^3+^ on anaerobic methane oxidation were assessed by incubation experiments with anoxic Black Sea water containing ANME-1b. Incubation experiments with ^13^*C*-methane and AQDS showed a stimulating effect of AQDS on methane oxidation. Fe^3+^ enhanced the ANME-1b abundance but did not substantially increase methane oxidation. Sodium molybdate, which was added as an inhibitor of sulfate reduction, surprisingly enhanced methane oxidation, possibly related to the dominant abundance of *Sulfurospirillum* in those incubations. The presented data suggest the potential involvement of ANME-1b in AQDS-enhanced anaerobic methane oxidation, possibly via electron shuttling to AQDS or via interaction with other members of the microbial community.

## 1. Introduction

Methane is a potent greenhouse gas (warming potential 34 times greater than CO_2_ (Forster et al. 2007), and its atmospheric concentrations is rapidly increasing [1]). There is a large and continuous production of methane in anaerobic marine sediments by methanogenic archaea. However, most of this methane is converted into carbon dioxide by oxidation, when methane is still in the sediment. It is estimated that marine anaerobic methane oxidizers consume 70–300 Tg CH_4_ year^−1^, reducing the atmospheric methane budget by 10–60% [2,3]. The methane that is not oxidized in the sediments, gets released into the water column, via diffusion or bubbling. From there, it can be emitted into the atmosphere. Methane oxidation in the water column can also (partially) consume this methane and thus forms an additional filter to prevent methane emission from marine systems.

To be thermodynamically favorable, anaerobic methane oxidation (AOM) needs to be coupled to the reduction of another compound. In marine settings, this compound is generally sulfate, and anaerobic methane oxidation is typically performed by a consortium of anaerobic methane-oxidizing archaea (ANME) and sulfate-reducing bacteria (SRB) [4]. Methane oxidation coupled to sulfate reduction yields a Gibbs free energy yield of only −17 kJ mol^−1^, which is near the minimum requirement for life, possibly being one of the factors explaining the slow growth rates of anaerobic marine methane oxidizers (doubling times 2–7 months [5,6]. Theoretically, methane oxidation coupled to the reduction of other compounds, such as Fe^3+^, nitrate, or nitrite, has a substantially higher energy yield [7]. Metal-oxide dependent AOM by ANME has been detected in enrichment cultures [8], but despite the thermodynamic advantages, observations of AOM not coupled to sulfate reduction in natural situations have been scarce. Egger et al. (2014) demonstrated iron oxide-mediated AOM was likely to occur in sediments of the Bothnian Sea (Northeast Baltic), but the microorganisms involved were not identified [9]. Scheller et al. (2016) showed that ANME were capable of performing methane oxidation with Fe^3+^ and 9,10-anthraquinone-2,6-disulfonate (AQDS), which was used as a humic acid analog [10]. Iron-mediated AOM, catalyzed by humic substances, was also detected by Valenzuela et al. (2019) [11]. Bai et al. (2019) also showed nitrate-reducing ANME were capable of using AQDS as an electron acceptor [12].

ANME oxidize methane via a reversed methanogenesis pathway (e.g. [13,14]). Three clades of ANME including several subclasses are recognized, all related to different groups of methanogens, namely ANME-1, ANME-2, and ANME-3 [13,15,16,17]. All clades are regularly found in syntrophy with sulfate-reducing bacteria (SRB), but whereas ANME-2 and ANME-3 are generally found in aggregates together with SRB cells, ANME-1 are often observed as single cells or loose aggregates [18]. The mechanism behind the ANME–SRB syntrophy is still under debate. A relationship based on the exchange of reaction products has been proposed [18,19,20,21], but other studies also suggested direct interspecies electron transfer [22,23]. ANME has been shown to be capable of forming intracellular wiring, creating a cell-to-cell connection that could allow a direct shuttling of electrons [22,23,24,25]. In this regard, the electron shuttling to abiotic particles such as oxidized metals or AQDS has only been observed in ANME of the clade ANME-2 [10,12].

The Black Sea is rich in methane due to the release from numerous cold seeps, and despite active methane oxidation in the sediments, water column methane concentrations below the chemocline are ca. 10–15 µM [26,27]. ANME-1 have been detected in the Black Sea water column before [28,29,30,31]. The ^13^C-depleted stable carbon isotopic composition of a- and monocyclic biphytanes derived from the characteristic membrane lipids of ANME-1 revealed that these archaea actively consume methane in the water column [29]. ANME have been suggested to play a major role in decreasing water column methane concentrations [31]. Although ANME-1 have often been observed in environments low in sulfate, and without a syntrophic SRB partner, their methane oxidation pathway independent of sulfate reduction remains unknown [32]. Previous studies have suggested a decoupling between AOM and sulfate reduction in the Black Sea water column, as no substantial stable carbon isotope depletion of SRB phospholipid fatty acid could be detected [29]. As AOM coupled to the reduction of alternative electron acceptors, such as humic substances or Fe^3+^, has a much higher (theoretical) energy gain than sulfate-mediated AOM, the availability of these electron acceptors could theoretically make AOM more thermodynamically favorable for ANME-1b [7].

To explore the metabolic versatility of ANME, the ANME-1b subgroup present in the anoxic water column of the Black Sea was studied. Suspended particulate matter (SPM) from the water column was collected and used for incubation studies with ^13^CH_4_, exploring the response of the microbial community to AQDS and Fe^3+^ in the presence of sodium molybdate (an inhibitor of sulfate reduction). The ^13^CO_2_ concentration was followed over time as a measure for methane oxidation. The microbial diversity at the end of the incubation experiments was analyzed by 16S rRNA gene sequencing to assess changes in the community composition under different conditions.

## 2. Materials and Methods

### 2.1. Sample Collection

Sampling was performed during cruise 64PE444 on R/V *Pelagia* in August 2018 at station 42° 53.8’ N 30° 40.7’ E. The conditions in the water column at the moment of sampling are shown in Fig. S1. Water samples were taken using a conductivity-temperature-density (CTD) system equipped with Niskin sampling bottles. Samples for nutrient analysis were collected directly after CTD recovery. A constant N_2_ flow during CTD sampling was used to retain anoxic conditions. N_2_ flushed pressure bottles (1 L) were filled with water collected at 1000 m depth by piercing the butyl stoppers with a needle.

Water column SPM from a depth of 1000 m was collected onto GF75 pore size 0.3 µm glass fiber filters (Advantec, Dublin, CA, USA) using a McLane WTS-LV in situ pump (McLane, East Falmouth, MA, USA), completed with a special filter head for anoxic sampling. Pumps were left in the water column to filter for 6 h. After pump recovery, the filter heads were transported to an anoxic glove bag, which was flushed with N_2_ three times before the overlying anoxic water was removed from the filters. Filters were then transferred to 1 L glass bottles filled with anoxic water collected from 1000 m, closed, and stored in the dark at 4–10 °C for 60 days until incubations were set up in the laboratory. Another filter was directly stored at −80 °C for analysis of the in situ microbial community. Water column samples for nutrient and DNA analysis were collected as described in Sollai et al. (2019) and Suominen et al. (2020) [30,33].

### 2.2. Incubations with Suspended Particulate Matter

To set up the incubation experiments, water column SPM was retrieved by scraping off the top layer of the glass fiber filters under anoxic conditions inside an anaerobic glove bag (Sigma Aldrich, St. Louis, MI, USA) under N_2_ atmosphere, and subsequently resuspended in 1 L of anoxic artificial seawater (commercially available mixture of sea salts, Sigma Aldrich, containing 28 mM SO_4_^2^^−^ but no sulfide) in incubation bottles of 1.2 L. All media in the anoxic bottles was boiled and bubbled with nitrogen for 20 min to remove residual oxygen, after which the bottles were closed, crimp sealed, and the headspace was flushed and exchanged with N_2_ gas using a GRInstruments (Wijk bij Duurstede, the Netherlands) automatic gas exchanger. Due to the used method, fibers of the filter were present in the incubation bottles. ^15^*N*-ammonium chloride (0.016 g) was also added for stable isotope activity measurements, but in the end, ^15^N incorporation was not measured. 10 mL ^13^CH_4_ (99% labeled; Sigma-Aldrich, St. Louis, MI, USA, resulting in a methane concentration of 500 µM) was added in order to follow methane-derived ^13^C over the course of the experiments. Depending on the type of incubation, 4.1 g sodium molybdate (Sigma-Aldrich), 0.03 g iron(III) citrate (Sigma-Aldrich), or 1.65 g anthraquinone-2,7-disulfonic acid disodium salt (AQDS, TCI Chemicals, Tokyo, Japan) or a combination of these (Table S1) was added to the medium. Autoclaved artificial seawater was used as an abiotic control to assess abiotic variations and instrument variability of the measured parameters. All experiments were performed in duplicate. The bottles were incubated in the dark at 10 °C for 58 days. Every 14 days, the bottles were shaken, and headspace gas was withdrawn for analysis. At the termination of the incubations, 10 mL of the medium was collected for nutrient analysis, stored at −20 °C until analysis, and processed as previously described [30]. The remaining medium was filtered over 0.3 µm GF75 filters (Advantec, Dublin, CA, US) for DNA analysis and was stored at −80 °C.

### 2.3. ^13^CO_2_ Analysis

^13^*C*-labeled carbon dioxide concentrations in the headspace of the incubation bottles were measured using a gas chromatograph (GC) equipped with a mass spectrometer (MS) (Agilent, Santa Clara, CA, USA, 7890B GC with 5975C MSD) in analytical triplicates. To study and compare the relatively small production or consumption of these compounds in the different incubation experiments with slightly different starting concentrations, the data of each individual incubation bottle was normalized on the starting value (*t*_0_) as 100%.

### 2.4. DNA Extraction and Analysis

DNA was extracted from the filters using the PowerSoil DNA extraction kit (MoBio Laboratories, Carlsbad, CA, USA) and stored at −80 °C until further analysis. The general 16S rRNA archaeal and bacteria primer pair 515F and 806RB targeting the V4 region [34] were used for the 16S rRNA gene amplicon sequencing and analysis, as described in Besseling et al. (2018) [35]. PCR products were gel purified using the QIAquick Gel-Purification kit (Qiagen, Hilden, Germany), pooled, and diluted. Sequencing was performed by the Utrecht Sequencing Facility (Utrecht, the Netherlands), using an Illumina MiSeq sequencing platform. Analysis of the 16S rRNA gene amplicon sequences was performed with the Cascabel pipeline [36], including quality assessment by FastQC [37], assembly of the paired-end reads with Pear [38], and assign taxonomy (including pick representative set of sequences with ‘longest’ method) with blast by using the Silva 128 release as reference database (https://www.arb-silva.de/). For analysis purposes, only species with a relative abundance greater than 0.001 were assumed significant. For tables and figures, results of duplicate bottles of the same treatment were averaged. The 16S rRNA amplicon reads (raw data) have been deposited in the NCBI Sequence Read Archive (SRA) under BioProject ID PRJNA605700.

### 2.5. Quantitative PCR 16S rRNA Gene

16S rRNA gene copies were quantified using quantitative PCR (qPCR) with the same primer pair as used for amplicon sequencing (515F, 806RB) on a Rotor-Gene 6000 (Corbett Research, Mortlake, Australia). The qPCR reaction mixture (25 µL) contained 0.5 U of Phusion High-Fidelity DNA Polymerase (Thermo Scientific), 1× Phusion HF Buffer, 0.2 µM of each dNTP, 20 μg of BSA, 0.6 pmol µL^−1^ of both primers, 0.5× EvaGreen dye (0.625 µM) in aqueous solution (Biotium, Hayward, CA, USA) and AccuGENE Molecular Biology Water (Lonza, Basel, Switzerland). The cycling conditions for the qPCR reaction were as follow: initial denaturation 98 °C for 30 s, 45 cycles of 98 °C for 10 s, 50 °C for 20 s, followed by fluorescence data acquisition, 72 °C for 30 s, and 80 °C for 25 s. Specificity of the reaction was tested with a gradient melting temperature assay, from 55 °C to 95 °C with 0.5 °C increments of 5 s apiece. The qPCR reactions were performed in duplicate with standard curves encompassing a range from 10^1^ to 10^7^ molecules µL^−1^. qPCR efficiency for the 16S rRNA gene quantification was 100% with R^2^ = 0.996. For quantification of microbial groups, the assumption that all microorganisms of the microbial community contained a single 16S rRNA copy in their genome was made, which has been confirmed by genome analysis for the ANME-1 group [25].

## 3. Results

The microbial community of the deep Black Sea water column was studied during incubation experiments, specifically focused on the response to additions of different electron acceptors and their effect on anaerobic methane oxidation. The methanotrophic activity in the incubations was assessed by the addition of ^13^*C*-labeled methane, followed by the analysis of ^13^CO_2_ concentrations over time.

### 3.1. Water Column Physicochemical Conditions and In Situ Microbial Community

**Figure 1:**
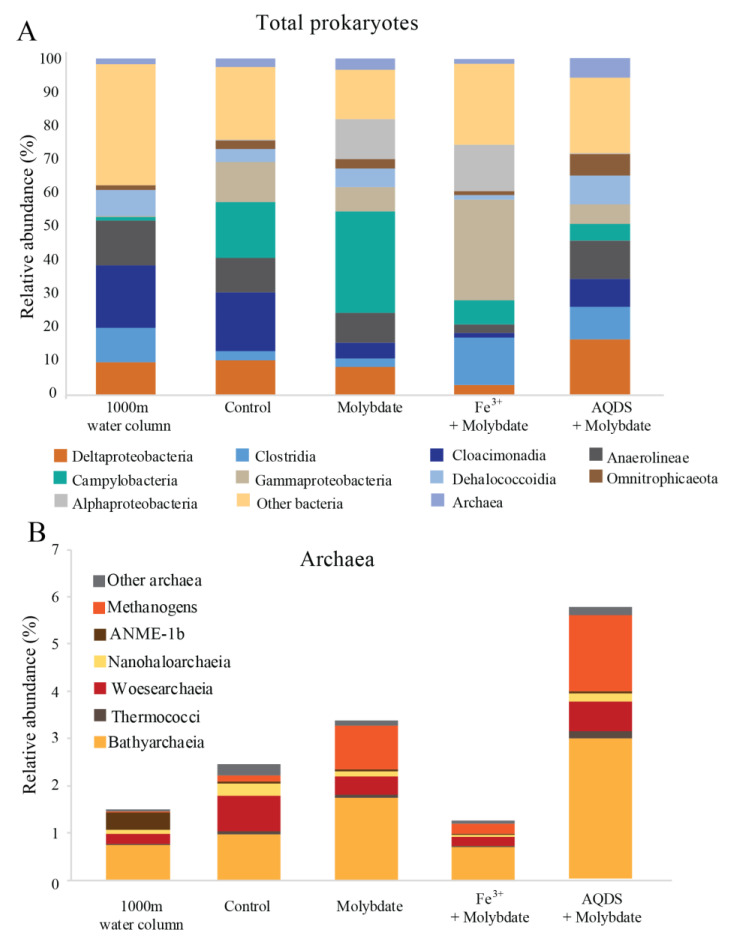
Total prokaryotic (**A**) and archaeal (**B**) diversity in Black Seas water from 1000 m depth and at the end of the various incubation experiments, expressed as percentage of total 16S rRNA gene reads. Methanogens defined here as all species belonging to the Methanomicrobia, Methanococci, Methanobacteria, Methanomassiliicoccales, and Methanofastidiosales, excluding any anaerobic methane oxidizing archaea (ANME). Values for duplicate bottles are averaged, except for the 1000 m sample, for which only one sample was used.

**Figure 2:**
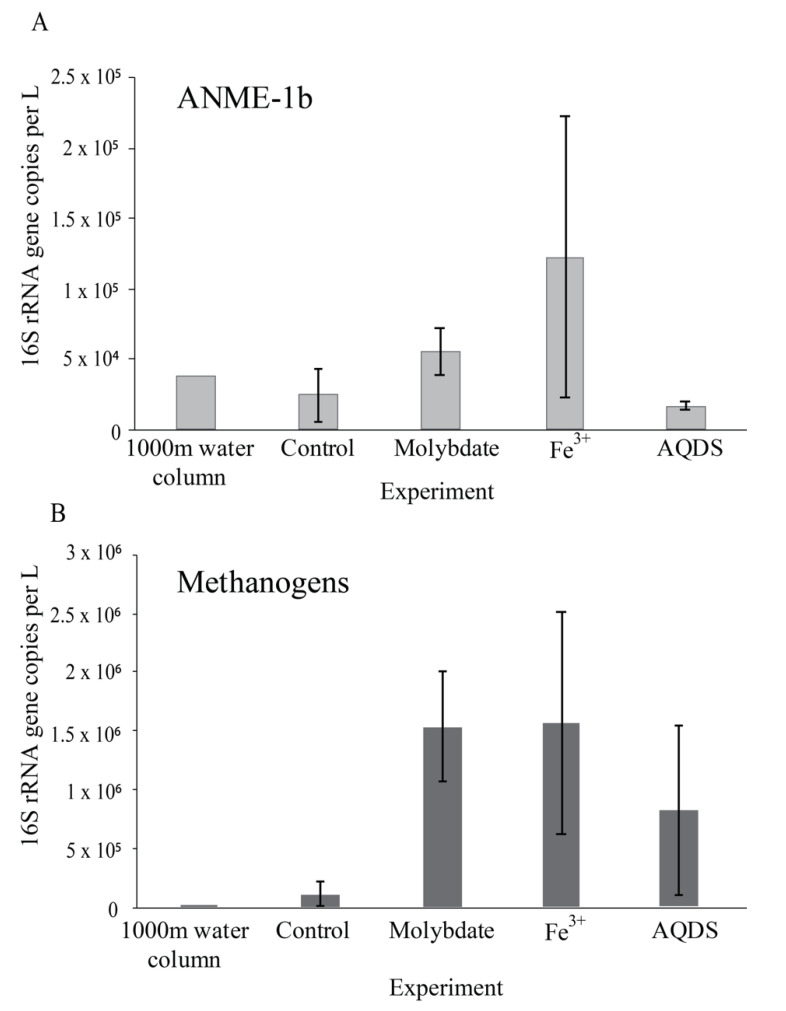
Abundance of ANME-1b (**A**) and methanogens (**B**); defined here as all species belonging to the Methanomicrobia, Methanococci, Methanobacteria, Methanomassiliicoccales, and Methanofastidiosales, (excluding any ANME) in natural Black Sea water from 1000 m depth and at the end of the various incubation experiments, in 16S rRNA gene copies per L. Values for duplicate bottles are averaged, except for the 1000 m sample, for which only one sample was used. Notice the different scale on the y-axis.

The Black Sea water column is over 2000 m deep and permanently stratified, with, at the studied station, an oxycline around 75 m depth (Figure S1). Water was sampled at 1000 m depth. The sulfate concentration at this depth was 17 mM and the nitrite concentration 21 nM (Figure S1). Diversity estimates based on 16S rRNA gene amplicon sequencing at 1000 m depth showed that the archaeal abundance was 2 × 10^5^ copies per L (i.e., 1.5% of the total 16S rRNA reads; Figure 1). 21% of the archaeal 16S rRNA reads were classified as ANME-1b (0.3% of the total 16S rRNA reads; Table 1 and Table S2; Figure 2), corresponding to 4 × 10^4^ copies per L. No known methanotrophs other than ANME-1b were detected in the library of 16S rRNA reads from 1000 m depth (average 190,000 reads per sample; Table S2). The abundance of methanogenic archaea (defined as 16S rRNA gene reads assigned to the Methanomicrobia, Methanococci, Methanobacteria, Methanomassiliicoccales, and Methanofastidiosales, but excluding ANME) amounted 4 × 10^3^ copies per L (2% of the archaeal 16S rRNA gene reads; Table 1 and Table S2; Figure 1 and Figure 2). 16S rRNA gene reads attributed to bacteria were closely related to Cloacimonadia (2 × 10^6^ copies per L, 19% of total 16S rRNA reads), Dehalococcoidia (9 × 10^5^ copies per L, 7.7%), Deltaproteobacteria (1 × 10^6^ copies per L, 9.8%, of which 5 × 10^5^ copies per L falling into SEEP-SRB cluster and 6 × 10^5^ copies per L to *Desulfatiglans* spp.), plus many other, less abundant groups (36% ‘other bacteria’; Figure 1).

### 3.2. Abiotic and Control Incubations

**Figure 3:**
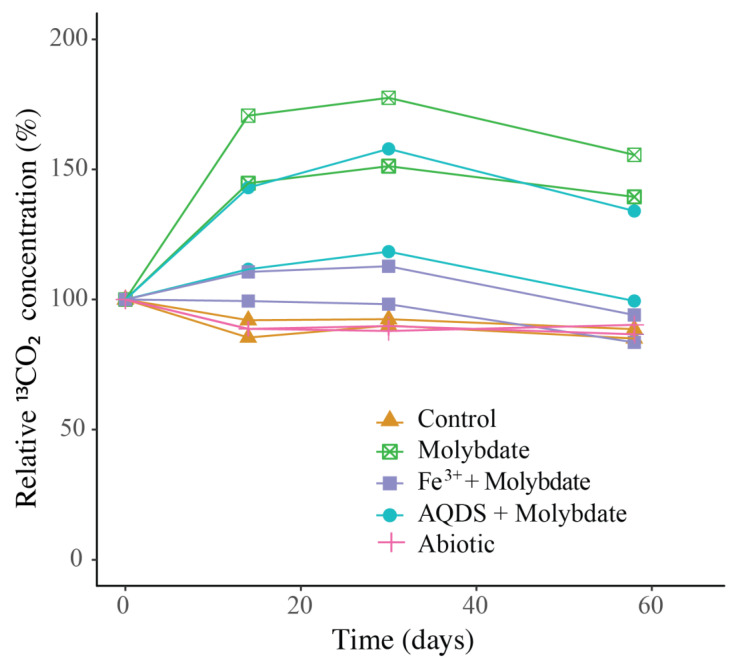
Change over time of ^13^CO_2_ in the incubation experiments, normalized to the concentration at *t_0_* (*t_0_* = 100%). The duplicate incubation bottles of each experiment are both shown individually. Actual concentrations are shown in Figure S3.

^13^CO_2_ concentrations in the abiotic incubations (artificial seawater with added ^13^CH_4_) and control (artificial seawater with added ^13^CH_4_ and microbial matter from the SPM) remained constant over the course of the experiment after a small initial decrease (Figure 3). The ANME-1b abundance in the control incubations was 2 × 10^4^ copies per L, corresponding to 1.4% of the archaeal 16S rRNA gene reads. No other microorganisms known to be capable of methane oxidation were detected. Reads assigned to methanogenic archaea made up 1 × 10^5^ copies per L (6.6% of the archaeal 16S rRNA gene reads; Table 1; Figure 2). The bacterial community was similar to that of the water column at 1000 m depth (Figure 1 and Figure S2), except for a higher relative abundance of Campylobacteria (1 × 10^7^ copies per L, 16% of total 16S rRNA reads; Figure 1) and Gammaproteobacteria (9 × 10^6^ copies per L, 12%; Figure 1). The genus *Sulfurimonas*, belonging to the Campylobacteria, comprised 5 × 10^6^ copies per L, corresponding to 7% of the 16S rRNA reads, the genus *Sulfurospirillum* (Campylobacteria) 5 × 10^6^ copies per L (6%; Table S2), *Desulfatiglans* (Deltaproteobacteria) 2 × 10^6^ copies per L (3%), *Fusibacter* (Clostridia) 1 × 10^6^ copies per L (2%) and SEEP-SRB (Deltaproteobacteria) 4 × 10^5^ copies per L (1%; Table 1 and Table S2). Each of these bacterial groups, except for SEEP-SRB, was more abundant in the control incubations than in the water column (Table 1; Figure S2).

### 3.3. Incubations with Sodium Molybdate

Sodium molybdate was used as an inhibitor of sulfate reduction in a subset of the incubation experiments, both with and without the addition of alternative electron acceptors (overview available in Table S1). In the incubation with only sodium molybdate, an increase of 65% in the ^13^CO_2_ concentration was observed from day 0 to day 30, after which the concentration slightly decreased (Figure 3 and Figure S3). The abundance of ANME-1b was 6 × 10^4^ copies per L in the molybdate incubation, which corresponded to a relative abundance of 1% of the archaeal 16S rRNA gene reads (Table 1; Figure 2). The abundance of methanogenic archaea increased substantially to 1.5 × 10^6^ copies per L (27% of the archaeal 16S rRNA reads; Table 1; Figure 2). Archaea of the Bathyarchaeia were relatively abundant compared to other archaea (3 × 10^6^ copies per L, 52% of archaeal 16S rRNA reads; Figure 1). The total 16S rRNA gene reads were strongly dominated by reads attributed to Campylobacteria (5 × 10^7^ copies per L, 31%), specifically of the genus *Sulfurospirillum* (5 × 10^7^ copies per L, 27%; Table 1 and Table S2). Members of the Deltaproteobacteria *Desulfatiglans* comprised 1 × 10^7^ copies per L (7%; Table 1 and Table S2), the *Sulfurimonas* 4 × 10^6^ copies per L (2%) and SEEP-SRB 9 × 10^5^ copies per L in the incubations with molybdate only (1%; Table 1 and Table S2).

### 3.4. Incubations with Sodium Molybdate and Soluble Fe^3+^ Complexes

The ^13^CO_2_ concentration in the Fe^3+^ amended incubations showed a slight increase during the experiment (on average 5%; Figure 3 and Figure S3) although the difference with the starting concentration was small. The abundance of ANME-1b was 1 × 10^5^ copies per L (1.3% of the archaeal reads; Table 1; Figure 2). The abundance of methanogenic archaea was 2 × 10^6^ copies per L (17% of the archaeal 16S rRNA gene reads; Table 1; Figure 2). Gammaproteobacteria dominated the Fe^3+^-amended incubations (2 × 10^8^ copies per L, 30% of total 16S rRNA reads, dominated by Vibrionales; Figure 1). The abundance of *Sulfurospirillum* was 4 × 10^7^ copies per L (5%, Table 1 and Table S2), the abundances of *Desulfatiglans*, *Fusibacter* and *Sulfurimonas* were 1–2 × 10^7^ copies per L for all three genera (2% of the 16S rRNA gene reads; Table 1 and Table S2).

### 3.5. Incubations with Sodium Molybdate and AQDS

The addition of AQDS to the incubations resulted in an increase of 38% in ^13^CO_2_ concentrations over the course of the experiment (Figure 3 and Figure S3). The abundance of ANME-1b at the end of the incubation was 2 × 10^4^ copies per L (1% of archaeal 16S rRNA reads; Table 1; Figure 2). The abundance of methanogenic archaea was 8 × 10^5^ copies per L (30% of archaeal reads; Table 1; Figure 2). The Bathyarchaeia made up just over half of the archaeal 16S rRNA reads (1 × 10^6^ copies per L, 52%; Figure 1). The 16S rRNA gene reads of bacteria were assigned to several major groups, each making up 5–18% of the community, with the Deltaproteobacteria being the most abundant (18%; Figure 1). The Deltaproteobacteria were dominated by the genus *Desulfatiglans* (6 × 10^6^ copies per L, 13% of 16S rRNA reads; Table 1 and Table S2). The abundance of the genus *Fusibacter* was 3 × 10^6^ copies per L (7%; Table 1 and Table S2). The abundance of the SEEP-SRB cluster was 8 × 10^5^ copies per L (representing 2% of 16S rRNA reads; Table 1 and Table S2). *Sulfurimonas*, *Sulfurospirillum*, and *Sulfurovum* abundances were 2 × 10^6^, 3 × 10^5^, and 2 × 10^5^ copies per L, respectively (representing 3, 0.6 and 0.4% of 16S rRNA reads, respectively; Table 1 and Table S2).

## 4. Discussion

Most commonly, marine AOM is coupled to sulfate reduction via a syntrophic relationship between ANME and SRB, based on the exchange of electrons or reaction intermediates [4]. In the past decade, several alternative electron acceptors for marine methane oxidation were proposed to be used by ANME, such as nitrate [39], iron and manganese [40], and humic substances [7,10,41]. ANME-1 are often found in the vicinity of SRB, but unlike ANME-2, they are found to be only loosely associated, not tightly aggregated [42,43], which raises the question if ANME-1 performs anaerobic methane oxidation independent of SRB and sulfate reduction. Here, this question was addressed by performing incubation experiments with alternative electron acceptors, using suspended particulate matter collected from the anoxic Black Sea water column which is naturally relatively rich in sulfate (17 mM, Figure S1) and where ANME-1b is present in the deep waters (4 × 10^4^ ANME-1b 16S rRNA gene copies per L at 1000 m; Figure 2; Table 1). No other organisms known to perform methane oxidation were detected at 1000 m depth. Therefore, we assume that AOM at this depth in the water column and in the incubation experiments is performed by ANME-1b, allowing us to test the electron donor preferences of ANME-1b in an incubation setup.

### 4.1. Enhanced Methane Oxidation by ANME-1b in Molybdate and in AQDS Incubations

In the control incubations, which contained the same sulfate-containing artificial seawater, SPM, and ^13^CH_4_ as the other experiments, no increase in the ^13^CO_2_ concentration over time was observed. Possibly, methane oxidation did not occur, or at too low rates to observe over the 60-day experiment. Methane oxidation rates by ANME in the Black Sea were previously reported to be 0.5–7 nmol L^−1^ day^−1^ at 1000 m [29]. The highest ^13^CO_2_ production rates observed in our experiments were much lower than would be expected at those methane oxidation rates (Figure S3). Potentially, the change in conditions from the water column to the incubation bottles affected the activity and methane turnover rate of ANME-1b. A change in pressure is known to affect both ANME and SRB abundance and activity [44]. Another possibility would be that ^13^CO_2_ is produced by ANME-1b but is simultaneously consumed by methanogens, SRB, or other microbial groups, and therefore no increase is detectable in the gas headspace. As ^13^C incorporation in the biomass was not measured, the fate of the labeled substrate cannot be fully determined.

The addition of only molybdate, and of molybdate plus AQDS, increased methane oxidation as seen by the detected increase of ^13^CO_2_ (Figure 3). We believe that the results found in these experiments are interesting to discuss in more detail, despite the low number of replicates per treatment. As sulfate reduction was assumed to be inhibited by the addition of sodium molybdate [45], it seemed surprising that methane oxidation could occur in the incubations with the addition of molybdate only and no alternative electron acceptor such as humic substances or Fe^3+^. Even if the inhibition of sulfate reduction was incomplete, and some sulfate reduction-coupled AOM would still have occurred, the methane oxidation rate in the molybdate-only experiments would not be expected to be higher than in the control incubations, that also contain sulfate and methane, but no inhibiting molybdate. It is therefore considered unlikely that sulfate-mediated AOM was responsible for the observed ^13^CO_2_ production, in both the molybdate only and the molybdate + AQDS incubation. More likely, an alternative form of AOM occurred when sulfate-mediated AOM was inhibited, possibly leading to more favorable energetic conditions for ANME (ΔG°′ sulfate-mediated AOM −17 kJ mol^−1^, ΔG°′ AQDS-mediated AOM −41 kJ mol^−1^ [10]). Although it is considered likely that AQDS was involved in AOM in the AQDS incubations, the question arises what was the role of molybdate, and whether it was molybdate rather than AQDS causing the enhanced ^13^CO_2_ production that was observed in those incubations, given the high ^13^CO_2_ production in the molybdate only incubation. Possibly, molybdate could have played a role as electron acceptor, as several studies have found specific bacteria are capable of the reduction of molybdate to molybdenum blue [46,47,48]. However, if it was purely the molybdate that caused the increase in methane oxidation rates in both the molybdate and in the AQDS incubations, the incubations with Fe^3+^ (containing molybdate) would have also been expected to show increased ^13^CO_2_ production, which was not the case (Figure 3). No substantial ^13^CO_2_ production was observed in the Fe^3+^ incubations, despite the relatively high abundance of ANME-1b compared to the AQDS incubations (Figure 2). The ANME-1b in the Fe^3+^ incubations may have been inactive or involved in methanogenesis rather than in methanotrophic pathways [49,50]. Possibly, the total 16S rRNA gene copies in the AQDS amended incubations, and thus also the ANME-1b abundance, was underestimated due to an inhibitory effect of humic substances on qPCR reactions, which is widely recognized [51,52] and could complicate the comparison between incubations with and without humic substances.

### 4.2. Potential Role of Sulfur Cycling Organisms

Several different sulfur processes have been linked to marine AOM, i.e., the reduction of sulfate [4], zero-equivalent sulfur [19], or polysulfides [53]. To explore the role of sulfur cycling in the incubation experiments, the organisms that were present and are known to be involved in sulfur cycling were studied. The only sulfur compounds that were added to the incubation experiments were sulfate, as part of the artificial seawater salts mixture, organic sulfur-containing microbial biomass of the SPM, and possibly small amounts of sulfur compounds that were present in the natural water column and that were transferred with the SPM that was used as the inoculum. We chose to add sulfate to all experiments, including those with molybdate, Fe^3+^, and AQDS, to retain similar conditions in all experiments, varying only the molybdate and alternative electron acceptor availability. Several microbial groups that are known to be involved in the sulfur cycle were abundant in the incubations. SRB were assumed to be inhibited by the addition of molybdate to all but the control incubations, but as sulfate consumption or sulfide production within the incubation experiments were not measured, it was not possible to determine whether sulfate reduction was completely inhibited.

The genera *Sulfurimonas*, *Sulfurospirillum,* and *Sulfurovum* increased drastically in abundance in the control incubations when compared to the water column (Table 1 and Table S2; Figure S2). This change could potentially be attributed to a bottle effect, in which the incubation conditions favor specific bacterial groups. These microorganisms have all been described to use sulfur (elemental S or polysulfides), thiosulfate, or sulfite as electron acceptor, and, for some strains, also as electron donor [54,55,56,57]. *Sulfurimonas*, and likely also *Sulfurovum* and *Sulfurospirillum*, are capable of oxidizing sulfide to produce sulfate as an end product and elemental sulfur and polysulfide as intermediate products [58,59,60]. The ability to autotrophically fix CO_2_ is likely widely present in different *Sulfurimonas*, *Sulfurospirillum*, and *Sulfurovum* species [56,58]. *Sulfurovum* and some *Sulfurimonas* strains are also capable of oxidizing sulfide and of using H_2_ as an electron donor [56,61]. It is unclear which compounds were cycled in the incubation experiments, and which affect the addition of molybdate had on these processes. In the control incubation, where no inhibitor of sulfate reduction was present, active sulfate reduction and sulfur cycling are expected to occur. As the named species are capable of CO_2_ fixation, they may have decreased the ^13^CO_2_ concentration in the control experiments, possibly diminishing the increase in headspace ^13^CO_2_ that was expected to occur, and that was taken as a measure of methane oxidation.

In the incubation with only molybdate, *Sulfurospirillum* sp. dominated the community (5 × 10^7^ copies per L, 27% of 16S rRNA reads; Table 1 and Table S2). As this was the main distinguishing factor between the molybdate and the other incubations, it could potentially be related to the enhanced AOM that was observed in these incubations (Figure 3). Recent research has suggested partner-independent AOM coupled to polysulfide reduction as a novel pathway of methane oxidation by ANME-1 [53], and potentially, *Sulfurospirillum* could produce these polysulfides. The production of polysulfides, however, requires the oxidation of sulfide, which was not added to our incubation experiments. It could be produced by the sulfur cycling organisms present (*Sulfurimonas*, *Sulfurospirillum, Sulfurovum*, SEEP-SRB) but it is unclear whether this could occur in the presence of molybdate, and why then specifically *Sulfurospirillum* became highly abundant in the molybdate incubations. In the AQDS incubations, *Sulfurospirillum* abundance was two orders of magnitude lower than in the molybdate only incubation (3 × 10^5^ copies per L, 0.6%; Table 1 and Table S2). In the Fe^3+^ amended incubation, the *Sulfurospirillum* abundance (4 × 10^7^ copies per L; Table 1) was comparable to the molybdate incubation, although the relative abundance was much lower (5%; Table S2).

Bacteria of the genera *Desulfatiglans* and *Fusibacter* became relatively more abundant in the incubations with AQDS (*Desulfatiglans* 13% of the 16S rRNA gene reads AQDS incubations, versus 3% in control incubations; *Fusibacter* 7% in AQDS incubations, vs. 2% in control incubations; Table S2), although this is not reflected in the absolute abundances (Table 1). *Desulfatiglans* sp. and *Fusibacter* sp. are known as strict anaerobes that can reduce sulfate, thiosulfate or sulfur, while oxidizing carbohydrates or other organic electron donors, such as AQDS [62,63,64]. Recently, *Desulfatiglans* sp. have been found to cooccur with ANME-1 and SEEP-SRB in estuarine sediments [65]. It is, however, unknown whether the sulfur compound reduction by *Desulfatiglans* sp. and *Fusibacter* sp. here could be coupled to AOM, and which sulfur compound they use here, as sulfate reduction is expected to be inhibited by the molybdate that was present in the incubations with AQDS. 

### 4.3. Potential Methanogenesis by Methanogens, ANME and Bathyarchaeota 

The abundance of methanogens in the molybdate and Fe^3+^ incubations was 10-fold higher than in control incubations (Table 1; Figure 2). Possibly, part of the produced CO_2_ in the incubations is converted back to CH_4_ by these methanogens, which would mean that the net produced ^13^CO_2_ is higher than was measured. It remains unclear what caused this increase in the methanogenic abundance.

Besides, ANME-1b has been shown to also be capable of methanogenesis, specifically under high H_2_ concentrations [65]. Although the concentration of H_2_ in the incubations was not measured, H_2_ may have built up as the consumption of H_2_ by SRB was likely inhibited by molybdate. Therefore, it is possible that ANME-1b switched to a methanogenic metabolism, producing methane rather than consuming it. In the molybdate and AQDS + molybdate incubations, a decrease in the ^13^CO_2_ concentration is observed after day 30 (Figure 3 and Figure S2). Possibly, this could be linked to such a metabolic switch.

The phylum Bathyarchaeota (i.e., the former Miscellaneous Crenarchaeotal Group) increased in abundance in all incubations supplemented with molybdate (i.e., from 8 × 10^5^ copies per L in the control to 3 × 10^6^, 5 × 10^6^ and 1 × 10^6^ copies per L in molybdate only, Fe^3+^ and AQDS, respectively; Figure 1). The Bathyarchaeota are known to be metabolically diverse, with subgroups potentially capable of methanogenesis [66], although organisms performing organic matter degradation and dissimilatory nitrogen and sulfur reduction are also present within this phylum [67,68,69,70,71]. The co-occurrence of Bathyarchaeota and ANME-1b in methane cold seeps has been suggested to be based on an indirect trophic relationship rather than a direct interaction [72] and the cause of the increase of the abundance of this phylum in the molybdate, Fe^3+^ and AQDS amended incubations remains unclear.

## 5. Conclusions

Overall, these results show that both molybdate and AQDS can stimulate methane oxidation in incubations with material from the Black Sea water column, containing ANME-1b. Enhanced methane oxidation by AQDS has been shown for ANME-2 in marine sediments [10] and also Valenzuela et al. (2017) observed an increase in ^13^CO_2_ production in sediment incubations with AQDS [73], but only when sulfate reduction was not inhibited and with barely detectable ANME-1 and ANME-3 abundances, making it difficult to assess which organisms were involved. To our knowledge, this is the first study showing that additions of molybdate, and of molybdate plus AQDS, are stimulating methane oxidation in incubations with water column SPM. We also believe this is the first study that suggests that ANME-1b is involved in an AQDS-stimulated AOM pathway. The mechanism behind the stimulating effects of molybdate and AQDS remains unclear. More research, including detailed measurements of the sulfur compounds in solution and gene expression analysis, is needed to reveal whether ANME-1b is indeed involved in AQDS-dependent AOM, whether sulfur compounds and a partner organism are involved, and what could be the role of this process in marine environments. 

## Figures and Tables

**Table 1:** Abundance (16S rRNA copies per L) of major species in the incubation experiments and the Black Sea water column.

Treatment	Total 16S rRNA Copies × µL^−1^	Archaea		Bacteria					
		ANME-1b	Methanogens *	*Desulfatiglans*	*Fusibacter*	SEEP-SRB	*Sulfurimonas*	*Sulfurospirillum*	*Sulfurovum*
1000 m water column	1.2 × 10^7^	3.8 × 10^4^	4.0 × 10^3^	5.6 × 10^5^	8.4 × 10^3^	4.5 × 10^5^	5.9 × 10^4^	2.0 × 10^4^	1.7 × 10^4^
Control	7.8 × 10^7^	2.4 × 10^4^	1.2 × 10^5^	2.1 × 10^6^	1.2 × 10^6^	4.4 × 10^5^	5.4 × 10^6^	4.6 × 10^6^	2.6 × 10^6^
Molybdate	1.7 × 10^8^	5.5 × 10^4^	1.5 × 10^6^	1.1 × 10^7^	3.1 × 10^6^	9.1 × 10^5^	4.1 × 10^6^	4.5 × 10^7^	4.0 × 10^5^
Fe^3+^	7.6 × 10^8^	1.2 × 10^5^	1.6 × 10^6^	1.2 × 10^7^	1.5 × 10^7^	1.4 × 10^6^	1.5 × 10^7^	3.5 × 10^7^	3.5 × 10^6^
AQDS	4.7 × 10^7^	1.7 × 10^4^	8.2 × 10^5^	6.1 × 10^6^	3.2 × 10^6^	8.2 × 10^5^	1.6 × 10^6^	2.6 × 10^5^	1.8 × 10^5^

* namely here Methanomicrobia, Methanococci, Methanobacteria, Methanomassiliicoccales and Methanofastidiosales, but excluding all anaerobic methane oxidizing archaea (ANME) groups.

## Supplementary Materials

The following are available online at https://www.mdpi.com/2076-2607/8/9/1288/s1, **Figure S1**. Environmental conditions in the Black Sea water column at the time of sampling, **Figure S2**. Abundance of major groups in the Black Sea water column (1000 m depth) and the control incubation experiment, in 16S rRNA copies per L-1, **Figure S3**. 13CO_2_ concentration in the headspace of the incubations with different electron acceptors, **Table S1**. Overview of the incubation experiments, **Table S2**. Total number of 16S rRNA reads per incubation, and the relative abundance (as % of the total 16S rRNA gene reads) of major species in the incubation experiments and the Black Sea water column. Values for duplicate bottles are averaged.
