# Supplementary material for: Assessing the Effect of Humic Substances and Fe(III) as Potential Electron Acceptors for Anaerobic Methane Oxidation in a Marine Anoxic System
Source: Microorganisms. 2020 Aug 24;8(9):1288. doi: 10.3390/microorganisms8091288 (PMC7564286; doi:10.3390/microorganisms8091288)
Supplement: Supplementary file 1 [file microorganisms-08-01288-s001.pdf]

Supplementary Materials

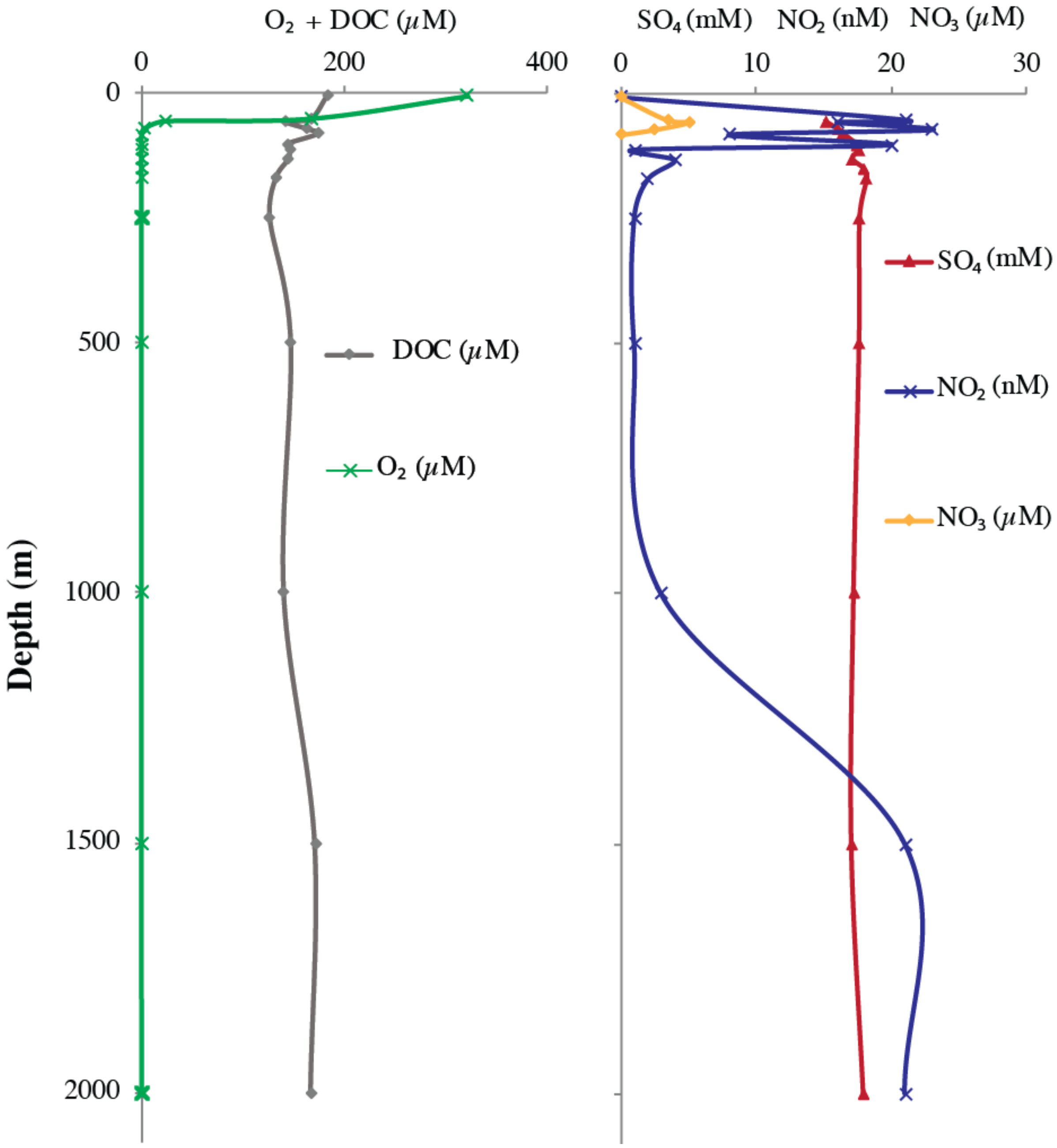

Figure S1. Environmental conditions in the Black Sea water column at the time of sampling.

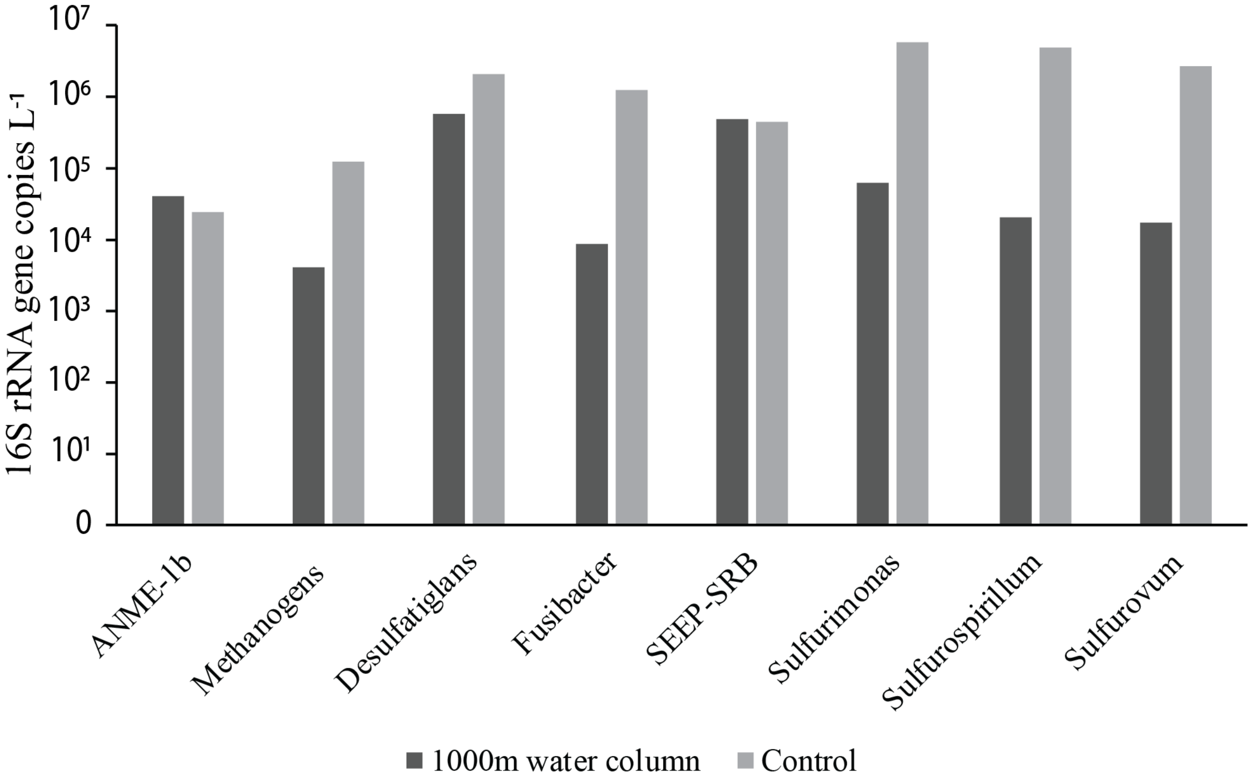

Figure S2. Abundance of major groups in the Black Sea water column (1000m depth) and the control incubation experiment, in 16S rRNA copies per L^{-1}. ANME – Anaerobic methane oxidizing archaea.

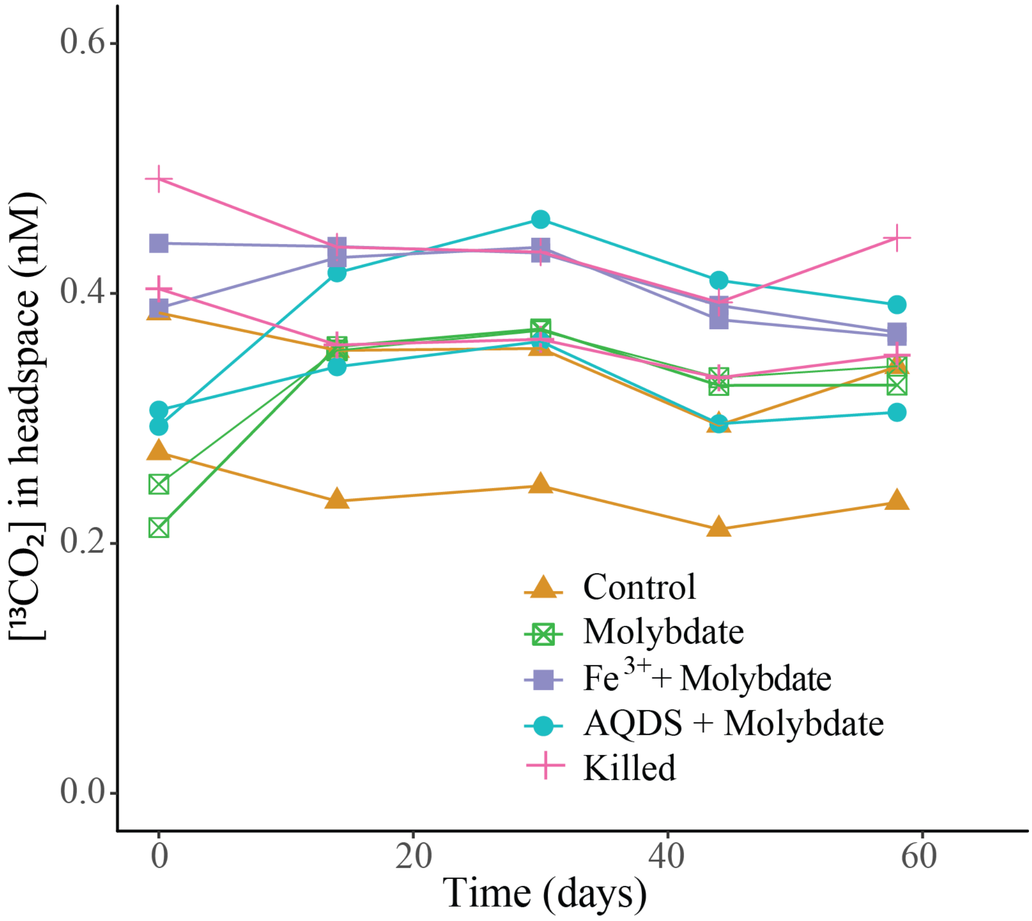

Figure S3. $^{13}CO_2$ concentration in the headspace of the incubations with different electron acceptors.

Table S1. Overview of the incubation experiments.

	**Artificial seawater** [1]	**$^{13}CH_4$**	**$^{15}NH_4$**	**Sodium Molybdate**	**Fe^{3+} citrate**	**AQDS**
Abiotic control	x	x				
Control	x	x	x			
Molybdate	x	x	x	x		
Fe^{3+} + molybdate	x	x	x	x	x	
AQDS + molybdate	x	x	x	x		x

1. Commercially available sea salts mixture (Sigma Aldrich).

Table S2. Total number of 16S rRNA reads per incubation, and the relative abundance (as % of the total 16S rRNA gene reads) of major species in the incubation experiments and the Black Sea water column. Values for duplicate bottles are averaged.

**Treatment**	**Total 16S reads per experiment**	**Archaea**		**Bacteria**					
		**ANME-1b**	**Methanogens[1]**	***Desulfatiglans***	***Fusibacter***	**SEEP-SRB**	***Sulfurimonas***	***Sulfurospirillum***	***Sulfurovum***
1000m water column	2.3×10^5	0.3	0.03	4.7	0.07	3.8	0.50	0.17	0.14
Control	2.1×10^5	0.03	0.15	2.6	1.5	0.56	6.9	5.9	3.3
Molybdate	2.4×10^5	0.03	0.92	6.8	1.8	0.54	2.4	27	0.2
Iron-oxides	1.7×10^5	0.02	0.22	1.6	1.9	0.19	1.9	4.6	0.5
AQDS	9.8×10^4	0.04	1.8	13	6.9	1.8	3.4	0.6	0.4

1. Namely here Methanomicrobia, Methanococci, Methanobacteria, Methanomassiliicoccales, and Methanofastidiosales, but excluding all ANME groups.
